# Mitochondrial genome divergence between beluga whales in Baffin Bay and the Sea of Okhotsk

**DOI:** 10.1080/23802359.2017.1318686

**Published:** 2017-04-28

**Authors:** Mikkel Skovrind, Jose Alfredo Samaniego Castruita, Mads Peter Heide-Jørgensen, Love Dalén, Eline Lorenzen

**Affiliations:** a Natural History Museum of Denmark, University of Copenhagen, Copenhagen K, Denmark;; b Greenland Institute of Natural Resources, Nuuk, Greenland;; c Department of Bioinformatics and Genetics, Swedish Museum of Natural History, Stockholm, Sweden

**Keywords:** Arctic, Monodontidae, Phylogeny, Western Greenland

## Abstract

The beluga whale is one of three endemic Arctic whales. The species is philopatric, and its migration patterns are passed from mother to calf. Management of the species is informed by the levels of genetic structuring among summer aggregation sites based on mitochondrial D-Loop data. To assess the levels of differentiation across the entire mitochondrial genome within belugas, we present a comparison between the first two complete mitochondrial genomes from opposite sides of their distribution range: Baffin Bay and the Russian Far East. Our analyses reveal that additional phylogenetic insights can be gained from expanding the genetic region analyzed. Further, we estimate the divergence time between the two mitochondrial genomes to be 0.469 MYA.

Here, we present the complete mitochondrial genome of a beluga whale (white whale, *Delphinapterus leucas*) from Baffin Bay. The beluga whale is a toothed whale belonging to the family Monodontidae, which also includes the narwhal *Monodon monoceros*. It has a discontinuous circumpolar distribution and is endemic to the Arctic region. Individuals can be up to 6.7 m long and are easily recognized by their white skin (Stewart & Stewart 1989). Mitochondrial DNA sequence information is of particular importance in the conservation of the beluga whale; the species is philopatric and does not re-colonize summer aggregation sites from which it has been extirpated (Brown Gladden et al. 1997). Previous studies have reported higher levels of genetic differentiation among summer aggregation sites based on mitochondrial D-Loop data than on up to 15 nuclear microsatellite markers (De March & Postma 2003). In light of this, other mitochondrial regions could be used to augment the 400–600 base pairs currently used for management purposes (O’Corry-Crowe et al. 2002; Turgeon et al. 2012; Meschersky et al. 2013).

A beluga whale reference mitochondrial genome from the Sea of Okhotsk, Russia, was recently published (Kim et al. 2017). The mitochondrial genome presented here is from Baffin Bay, and therefore represents the opposite end of the distribution range of the species. The analysis of this specimen may identify mitochondrial genomic regions of interest for further studies. The individual was sampled during the Inuit subsidence hunt in Qeqertarsuaq (69.237835, −53.526519) in western Greenland in April 2008 by staff from Greenland Institute of Natural Resources. The tissue sample is stored at the Natural History Museum of Denmark (ID number CGG_1_017647). The sequence is available from GenBank under accession number KY888944.

We extracted DNA from skin tissue using the Kingfisher Duo extraction robot and Cell and Tissue DNA Kit from ThermoFisher Scientific using the manufacturer’s protocol (ThermoFisher Scientific, Waltham, MA). Paired-end sequencing was performed on 180 base pair inserts using the Illumina HiSeq X platform (San Diego, CA). We assembled the mitogenome using a combination of MIRA 4.0.2 (Chevreux et al. 1999) and MITObim v.1.8 (Hahn et al. 2013), using the narwhal reference genome as a reference (Genbank accession: NC005279). We performed the annotation using the MITOS web service (Bernt et al. 2013) using default parameters. We performed a phylogenetic analysis using the 13 protein-coding regions across six closely-related toothed whale species and the beluga reference mitochondrial genome (Kim et al. 2017). The phylogenetic tree was constructed from the best tree among 100 independent runs in RAxML v.8.2 (Stamatakis 2014) using a GTR-GAMMA substitution model (Figure 1). Topology was verified by 100 bootstraps. The number of variable sites in each genomic region was calculated using FaBox 1.4.1 (Villesen 2007). Divergence time (*T*) between the two mitochondrial genomes was calculated as *T* = *K*/(2*υ*), using a mutation rate (*υ*) estimated for killer whales of 2.60 × 10^−3^ substitutions per site per million years (1.50–3.83 × 10^−3^) (Morin et al. 2008) and a pairwise differentiation (*K*) of 2.44 × 10^−3^ calculated as the number of variable sites per site.

**Figure 1.**
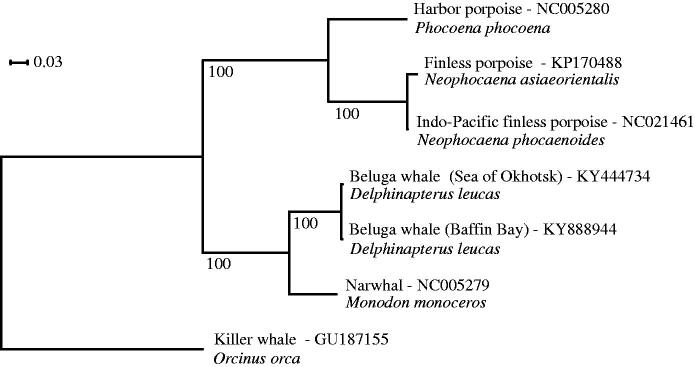
Phylogenetic tree of beluga whales from Baffin Bay and Sea of Okhotsk, and five related toothed whale species.

Our assembly yielded a 16,386 base-pair circular genome with gene regions in accordance with the findings of Kim et al. (2017). We found a total of 40 variable sites between the two beluga mitochondrial genomes, with 4, 2, 6 and 28 variable sites in the tRNA, rRNA, D-loop and protein-coding regions, respectively. The rRNAs had one variable site each and the protein-coding regions had between 1 and 5 variable sites (ND1:1, ND2:5, COX1:3, COX2:2, ATP8:1, ATP6:1, COX3:2, ND3:1 ND4L:1, ND4:2, ND5:4, ND6:3, CYTB:2), indicating that additional phylogenetic information is available in the mitochondrial genome relative to the D-Loop. We estimated a divergence time between the two mitochondrial linages of 0.469 (0.319–0.814) MYA, which is the same order of magnitude as the divergence time estimation of killer whale lineages (Morin et al. 2008).
